# Loss of PDPK1 abrogates resistance to gemcitabine in label-retaining pancreatic cancer cells

**DOI:** 10.1186/s12885-018-4690-1

**Published:** 2018-07-31

**Authors:** Dandan Li, John E. Mullinax, Taylor Aiken, Hongwu Xin, Gordon Wiegand, Andrew Anderson, Snorri Thorgeirsson, Itzhak Avital, Udo Rudloff

**Affiliations:** 1 Rare Tumor Initiative, Cancer for Cancer Research, National Cancer Institute, Building 10, Room 2B-38E, Bethesda, MD USA; 2 Sarcoma Department, Moffitt Cancer Center, Tampa, FL USA; 3 Thoracic & GI Oncology Branch, Center for Cancer Research, National Cancer Institute, Bethesda, MD USA; 4 Department of Surgery, University of Wisconsin School of Medicine and Public Health, Madison, WI USA; 5 Laboratory of Oncology, Center for Molecular Medicine and Department of Molecular Biology and Biochemistry, School of Basic Medicine, Yangtze University, Jingzhou, Hubei China; 6 Flow Cytometry Core, Hollings Cancer Center, Medical University of South Carolina, Charleston, SC USA; 7 Gilead Sciences, Foster City, CA USA; 8 Laboratory of Experimental Carcinogenesis, Center for Cancer Research, National Cancer Institute, NIH, Bethesda, USA; 9 St. Peter’s Hospital, Rutgers University, Robert Wood Johnson School of Medicine, New Brunswick, NJ USA

**Keywords:** Pancreatic cancer, Cancer stem cell, Label-retaining cancer cells (LRCC), PDPK1, Chemoresistance

## Abstract

**Background:**

Label-retaining cancer cells (LRCC) have been proposed as a model of slowly cycling cancer stem cells (CSC) which mediate resistance to chemotherapy, tumor recurrence, and metastasis. The molecular mechanisms of chemoresistance in LRCC remain to-date incompletely understood. This study aims to identify molecular targets in LRCC that can be exploited to overcome resistance to gemcitabine, a standard chemotherapy agent for the treatment of pancreas cancer.

**Methods:**

LRCC were isolated following Cy5-dUTP staining by flow cytometry from pancreatic cancer cell lines. Gene expression profiles obtained from LRCC, non-LRCC (NLRCC), and bulk tumor cells were used to generate differentially regulated pathway networks. Loss of upregulated targets in LRCC on gemcitabine sensitivity was assessed via RNAi experiments and pharmacological inhibition. Expression patterns of PDPK1, one of the upregulated targets in LRCC, was studied in patients’ tumor samples and correlated with pathological variables and clinical outcome.

**Results:**

LRCC are significantly more resistant to gemcitabine than the bulk tumor cell population. Non-canonical EGF (epidermal growth factor)-mediated signal transduction emerged as the top upregulated network in LRCC compared to non-LRCC, and knock down of EGF signaling effectors PDPK1 (3-phosphoinositide dependent protein kinase-1), BMX (BMX non-receptor tyrosine kinase), and NTRK2 (neurotrophic receptor tyrosine kinase 2) or treatment with PDPK1 inhibitors increased growth inhibition and induction of apoptosis in response to gemcitabine. Knockdown of PDPK1 preferentially increased growth inhibition and reduced resistance to induction of apoptosis upon gemcitabine treatment in the LRCC vs non-LRCC population. These findings are accompanied by lower expression levels of PDPK1 in tumors compared to matched uninvolved pancreas in surgical resection specimens and a negative association of membranous localization on IHC with high nuclear grade (*p* < 0.01).

**Conclusion:**

Pancreatic cancer cell-derived LRCC are relatively resistant to gemcitabine and harbor a unique transcriptomic profile compared to bulk tumor cells. PDPK1, one of the members of an upregulated EGF-signaling network in LRCC, mediates resistance to gemcitabine, is found to be dysregulated in pancreas cancer specimens, and might be an attractive molecular target for combination therapy studies.

**Electronic supplementary material:**

The online version of this article (10.1186/s12885-018-4690-1) contains supplementary material, which is available to authorized users.

## Background

Pancreatic ductal adenocarcinoma (PDAC) is an especially lethal disease with 53,070 new cases diagnosed last year and 41,780 deaths due to disease [1]. Its 5-year survival rate of 5–8% has not substantially changed over the last three decades and the American Association for Cancer Research (AACR) estimates pancreas cancer to rank second in cancer-related mortality in the U. S by the year 2020 [2]. Despite recent significant advances in the knowledge of the underlying molecular mechanisms in PDAC, meaningful long term survival remains elusive [3]. More than 80% of patients present with locally advanced or distant metastatic disease at time of diagnosis, which precludes operative extirpation and, therefore the only modality associated with longer term survival. These patients are thus relegated to palliative systemic therapies with the best combination of conventional cytotoxic chemotherapy for advanced pancreas cancer conferring a median survival estimate of less than 1 year [4, 5]. Given the dismal long term survival for the vast majority of patients with this disease, new therapeutic approaches in treatment of this disease are needed.

The cancer stem cell (CSC) theory holds that: 1) cancer arises from cells with dysregulated self-renewal mechanisms; and, 2) cancer is comprised of a heterogeneous mass of cells, a small fraction of which consists of stem-like progenitor cells that drive tumor growth and metastasis [6, 7]. The theory itself is a progression of Knudson’s two-hit hypothesis of carcinogenesis (initiation and promotion), though the origin of the cell lineage involved with initiation and promotion of neoplastic growth is different. A detailed pancreas cancer-specific stem cell phenotype-genotype association remains elusive, which is, in part, due to the different standards of definition and isolation of such cells but also due to an increased recognition of the inherent heterogeneity of the CSC fraction [8–12] While many groups have described cancer stem cells from multiple tissue sources using a variety of methods, these reported methods rely on cell surface moieties as a surrogate for the identification of these stem cells, but do not necessarily isolate CSCs in a manner reflective of their proposed function and hierarchy [12–15].

Almost 40 years ago ‘mutational selection’ in cancer was described and followed 3 years later by the first description of label retaining cells (LRC) and the ‘immortal strand hypothesis’ [16, 17]. Label-retaining cells (LRC) are associated with populations of cells enriched with adult tissue stem cells [18–21]. Many solid organ cancers develop in tissues found to harbor LRC and it is increasingly recognized that slowly cycling LRCC exhibit cancer stem cell and pluripotency traits representing a distinct subpopulation of the heterogeneous CSC pool [5, 22–26]. The clinical importance of the LRCC subpopulation has recently been demonstrated in a sentinel report of repopulation of residual tumors post-chemotherapy treatment with new cancer cells from this pool of cells [27]. Other reports have linked slowly cycling LRCC to disseminated tumor cells (DTC), relapse, and metastasis in cancer patients [28, 29]. Recently, we demonstrated that label-retaining cancer cells (LRCC) undergo asymmetric cell division, and represent a unique subpopulation of tumor-initiating stem-like cells with pluripotency gene expression profiles [20]. While early reports described fixed cells, which precluded downstream analysis, we recently published on such a method for the isolation of live tissue-derived LRC allowing for future assays dependent on live functioning cells. Using these methods it was shown that LRC do, in fact, undergo asymmetric cell division with non-random chromosomal cosegregation (ACD-NRCC) [20, 30].

The identification of LRCs in PDAC [i.e. pancreatic cancer-derived label retaining cancer cells (LRCC)] would offer a unique opportunity to study features of cancer stemness, in particular with regard to identifying vulnerabilities of this cell population knowledge which has remained elusive for the design of more effective therapies in pancreas cancer and drug development in general. Despite the ability to potentially impact sentinel events in cancer recurrence and progression, there is significant paucity in the understanding of selective molecular mechanisms in LRCC.

In the following report, we compare the transcriptome of LRCC and non-LRRC in pancreas cancer cell lines and identify perturbations unique to LRCC. Targeting one of the genes selectively upregulated in LRCC, 3-phosphoinositide dependent protein kinase-1 (PDPK1), we demonstrate that the phenotype resistance to chemotherapy in pancreatic cancer LRCC can be abrogated as a potentially novel treatment avenue against this difficult to treat cell population possibly guiding novel combination therapies in this lethal disease.

## Methods

### Cell culture

The cell lines MiaPaCa2 (ATCC, Manassas, VA, Cat. # ATCC-CRL-1420), Panc-1 (ATCC, Manassas, VA, Cat. # ATCC-CRL-1469), and were grown in DMEM medium supplemented with 10% FBS, 1% PenStrep, and 1% 200 mM L-glutamine (Gibco, Grand Island, NY). The Nor-P1 (Riken BioResource Research Center, Japan, Cat.# RBRC-RCB2139) cell line was grown in RPMI 1640 medium supplemented with 10% FBS, 1% PenStrep, and 1% 200 mM L-glutamine (Gibco, Grand Island, NY). Hereafter these media are considered “standard” media. Serum free media contained all elements with the exception of FBS and antibiotic free media contained all elements with the exception of PenStrep.

### Isolation of label retaining cancer cells

Cells were cultured in standard media until 80% confluency. One cell cycle before labeling, the media was changed to serum free media. Prior to labeling, the cells were lifted with 0.25% Trypsin (Gibco, Grand Island, NY) and resuspended in R-buffer (Invitrogen, Grand Island, NY) at a concentration of 5 × 10^6^ cells/100uL. Cy5-dUTP (GE Healthcare, Piscataway, NJ) was added at a concentration of 12uL/5 × 10^6^ cells. The cells were transfected using the Invitrogen Neon Transfection System using 1200 V for 20 milliseconds and 2 pulses. Immediately following transfection, the cells were placed in antibiotic free media and grown at 37 °C with 5% CO_2_ for one cell cycle. Following this brief culture, the cells were again lifted and sorted for Cy5-dUTP purity using a BD FACSAria II instrument (BD Biosciences, San Jose, CA). The Cy5-dUTP^**+**^ fraction was placed back into culture and expanded for 8 cell cycles, splitting cells at 70% confluency. Subsequently, the cells were sorted using a BD FACSAria II instrument (BD Biosciences, San Jose, CA). The Cy5-dUTP^**+**^ cells represent the label retaining cancer cells and the Cy5-dUTP^**−**^ cells represent the non-label retaining cancer cells. Cells were used immediately for downstream analyses.

### Gene expression analysis

Total RNA was isolated using Arcturus PicoPure RNA Isolation Kit (LifeTechnologies, Carlsbad, CA). The quality and quantity of RNA was assessed using the Agilent 2100 Bioanalyzer (Agilent Technologies, Wilmington, DE) and only total RNA with a RIN > 8 was amplified using the Illumina TotalPrep RNA Amplification Kit (Life Technologies, Carlsbad, CA). Following amplification of 200 ng total RNA, the biotin-cRNA was loaded onto an Illumina HT-12v4 BeadChip and data was obtained using the Illumina iScan device (Illumina, San Diego, CA). Raw data was exported from Illumina GenomeStudio to Agilent GeneSpring GX v11 for downstream expression analysis. Pathway analysis was performed using Ingenuity Pathway Analysis software. Results were validated using TaqMan qRT-PCR with primers specific to the microarray sequences which were obtained from Genecopoeia (Rockville, MD).

### Cell cycle analysis

Live cells were fixed using 70% EtOH following FACS sorting. The cells were washed twice using PBS and resuspended in 1 mL of staining solution which contained 10 ml of 0.1% (*v*/v) Triton X-100 (Sigma) in PBS, 2 mg DNase-free RNase A (Sigma), and 200 μl of 1 mg/ml PI (Sigma). Cells were incubated at 37 °C for 30 min and then immediately analyzed using the BD FACSAria II instrument.

### Cell proliferation and apoptosis assay

The IC50 dose of gemcitabine hydrochloride (Gemzar® Eli Lilly, Indianapolis, IN) was calculated for each cell line using the CellTiter-Glo® assay (Promega, Madison, WI), after exposure to a serial dilution of drug in a 96 well plate format for 72 h. Live cells were plated following FACS at a concentration of 3000 cells/well in 100uL standard media. Following incubation for 24 h at 37 °C with 5% CO_2_, the media was changed to standard media with the addition of the IC50 dose of gemcitabine hydrochloride for the given cell line. After 72 h cell proliferation was assessed using the CellTiter-Glo® assay with levels of untreated cells normalized to 100%. Additionally, apoptosis was evaluated at the same time using the Caspase-Glo®3/7 assay (Promega, Madison, WI).

### Exposure to gemcitabine following siRNA transfection

Cells were plated at a concentration of 3000 cells/well in 100uL media containing antibiotic free media with the addition of 0.3 μL RNAi Max transfection agent (Life Technologies, Carlsbad, CA) and 2 μL of 1 μM siRNA (GeneSolution, Qiagen, Valencia, CA) reconstituted in RNase free water. Following transfection for 48 h_,_ cells were then exposed to gemcitabine hydrochloride for 72 h and final cell viability and apoptosis were measured using the CellTiter-Glo® assay and Caspase-Glo®3/7 assay, respectively. Data analysis was performed using GraphPad Prism6 software. Drug response curves were created using a four-parameter equation fitting technique.

### Tissue microarray (TMA) composition

De-identified cancer tissues were confirmed to be pancreatic ductal adenocarcinomas based on pathology slide review at the National Cancer Institute. The analytic dataset included 144 specimens from the Iowa, Hawaii and Los Angeles Surveillance, Epidemiology, and End Results (SEER) Residual Tumor Registries pancreatic cancer tissue microarray (TMA) [31]. An additional commercial TMA (Biomax) with 40 matched tumor and normal pancreatic tissue specimens was used to compare PDK1 expression between tumor specimens and normal pancreatic tissue.

### Immunoblot and immunofluorescence analysis

Cancer cells were lysed with M-PER® Mammalian Protein Extraction Reagent (Cat#78501, ThermoScientific, Waltham, USA) plus Halt™ protease & phosphatase inhibitor cocktail (Cat#1861284, ThermoScientific, Waltham, USA). Protein concentration was determined via BCA analysis kit (ThermoScientific, Waltham, USA). For immunoblotting, proteins were transferred from 4 to 20% SDS/Polyacrylamide gels to nitrocellulose blotting papers via the iBlot®2 Gel Transfer Device (LifeTechnologies, Carlsbad, CA). The phospho-PDPK1 Ser241 antibody (Cat#3438), the phospho-AKT Ser473 antibody (Cat#9271), the AKT antibody (Cat#9272) and β-Actin antibody (Cat#4970, all Cell Signaling, Danvers, USA) were applied and bands were visualized via the Odyssey luminescence scanner (Li-Cor, Lincoln, USA). For immunofluorescence analysis, approximately 50,000 were centrifuged onto a glass slide with Rotofix 32 A centrifuge (Hettich Lab Technology, Tuttlingen, Germany) and fixed in 4% paraformaldehyde at 4 °C overnight. Cells were permeabilized in 0.25% TritonX-100 and blocked with 5% normal goat serum in PBS at room temperature in a humidified chamber for 2 h. Slides were incubated anti-phosphatidylinositol 3,4,5-trisphosphate (PIP3) (Cat#Z-P345, Echelon Biosciences Inc., Salt Lake City, UT) monoclonal antibodies. Alexa Fluor® 488 goat anti-mouse IgG (H + L) secondary antibody was then applied for 1 h at room temperature. Slides were mounted with Vectashield/DAPI (Vector Laboratories, Burlingame, CA). Images were captured using a Zeiss LSM 510 UV confocal microscope (Zeiss, Thornwood, NY).

### Immunohistochemistry and statistical analysis

Immunohistochemical staining for PDPK1 (HPA027376; Sigma Aldrich, St. Louis, MO) was performed by NDBio, Baltimore, MD. PDPK1 expression was evaluated semiquantitatively for expression levels via a four-tier scale (0 = negative; 1 = background; 2 = positive; 3 = strongly positive) and for cellular localization as having cytoplasmic PDPK1 expression, membrane PDPK1 expression, or a combination of both patterns. Evaluation of staining was carried out in a blinded fashion with respect to outcome and stage.

### Statistical analysis

Matched tumor and normal pancreatic tissues were compared using Wilcoxon matched-pairs signed rank test. Product-limit survival estimates were plotted using the Kaplan-Meier method with significance determined by log-rank test. Comparison of staining pattern (cytoplasmic vs membrane) with respect to histologic grade was performed using Fisher’s exact test.

## Results

### Pancreas cancer label retaining cancer cell (LRCC) isolation

**Fig. 1:**
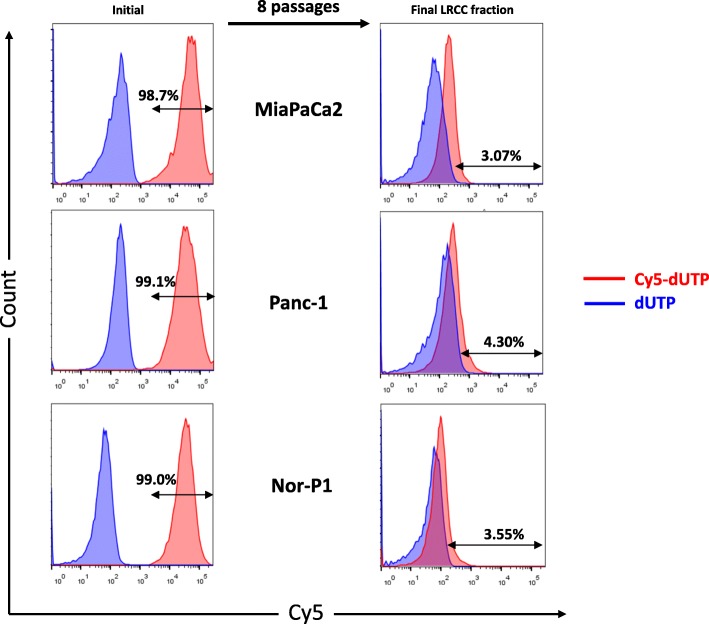
LRCC isolated from pancreatic adenocarcinoma cell lines. Flow cytometry measuring Cy5-dUTP labelled cell fraction (x-axis). The amount of LRCCs (measured by Cy5-dUTP positive fraction) after multiple passages is higher than mathematically expected for symmetric cell division

Label retaining cancer cells (LRCC) were isolated from the cell lines MiaPaCa2, Panc-1, and Nor-P1 following culture for a period equal to eight doubling times (Fig. 1). Following this expansion, approximately 0.4% of the cells would be mathematically expected to retain the label assuming symmetric division. The proportion of LRCC exceeded that which would be expected mathematically (0.4%) with 3.07, 4.30, and 3.55% measured LRCC fraction isolated for MiaPaCa2, Panc-1, and Nor-P1, respectively (*p* = 0.0119). This significant increase in the observed compared to the expected proportion of LRCC is consistent with previous observations of ours and others of non-stoichiometric division of genetic material during cell division suggestive of known asymmetric cell divisions of pluripotent cells with stemness features [20, 30].

### Responses to gemcitabine differ in LRCC and bulk cell population

**Fig. 2:**
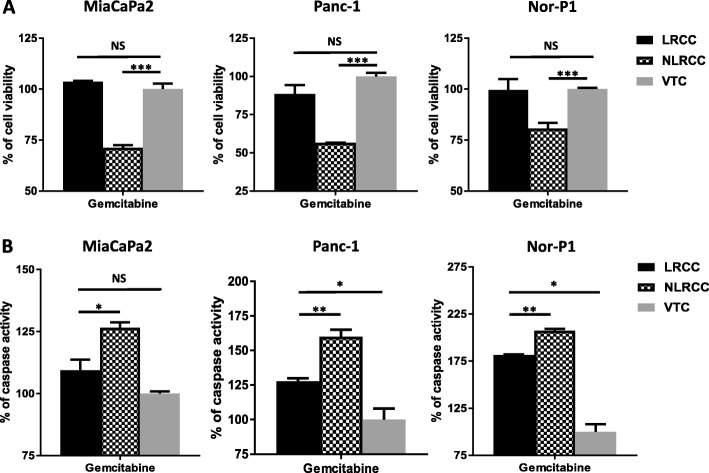
Responses in LRCC and NLRCC differ following exposure to gemcitabine. **a** LRCC resistance as measured by cell proliferation assay (normalized to vehicle-treated cells (VTC) after treatment with gemcitabine (GI50) for two doubling times in MiaPaCa2, Panc-1, and Nor-P1 cells (*p < 0.05, **p < 0.01, ***p < 0.001; paired *t*-test). **b** Activated caspase 3/7 assay measures reduced apoptosis in LRCC population upon exposure to gemcitabine compared to NLRCC

LRCC, non-label retaining cancer cells (NLRCC), and unsorted cells were exposed to the previously determined GI50 of gemcitabine of bulk cells of 33 nM (MiaPaCa2), 3 μM (Panc-1) and 3.3 nM (Nor-P1) for a period equal to two doubling times. After normalizing vehicle-treated cells (VTC) to 100%, relative proliferation after exposure to gemcitabine GI50 of the LRRC fraction was 103.7% for cell line MiaPaCa2, 88.5% for Panc-1, and 99.7% for Nor-P1, whereas survival in the NLRCC fractions decreased to 71.3, 56.6, and 80% compared to vehicle-treated cells (LRCC vs non-LRCC populations in MiaPaCa2 (*p* < 0.001), Panc-1 (*p* < 0.01), and Nor-P1 (*p* < 0.05) cells), following treatment with gemcitabine, respectively (Fig. 2a). In line with the genotoxic activity of gemcitabine, we examined levels of apoptosis in LRCC and non-LRCC populations as a possible mechanism of action for the observed difference in response to gemcitabine. Activated caspase 3/7 activity in the LRCC fraction of MiaPaCa2, Panc-1, and Nor-P1 was measured as 109.4, 127.7, and 181.5% compared to vehicle-treated control whereas apoptosis in the NLRCC fraction upon gemcitabine treatment increased to 126.6% (*p* < 0.05), 160.0% (*p* < 0.01), and 207.4% (*p* < 0.01), respectively (Fig. 2b). Apoptosis levels of LRCC and NLRCC subpopulations were normalized to caspase 3/7 levels of vehicle-treated cells.

### Resistance to gemcitabine is an indigenous feature of LRCC

**Fig. 3:**
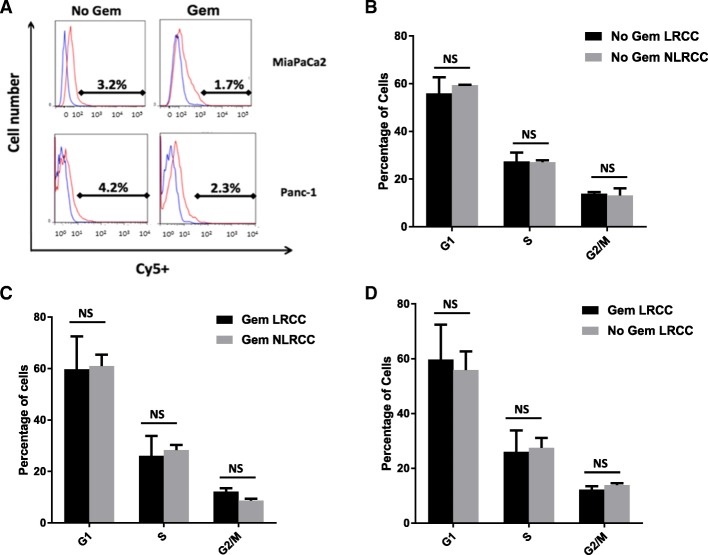
LRCC resistance is not due to increased proportion of the LRCC fraction or increased quiescence following exposure to gemcitabine. **a** Flow cytometry measuring Cy5-dUTP labelled cells (x-axis) are shown. Gemcitabine (at GI50 for individual cell line) was administered after 6 doubling times and measurements were taken after a total of 8 cycles. **b** Cell cycle distribution of LRCC and NLRCC cells without gemcitabine treatment, average of two cell lines of ≥2 independent experiments per cell line are shown. **c** Cell cycle distribution of gemcitabine treated LRCC and NLRCC cells, average of two cell lines of ≥2 independent experiments per cell line are shown. **d** Cell cycle distribution of gemcitabine treated and untreated LRCC cells, average of two cell lines of ≥2 independent experiments per cell line are shown

Next, we evaluated the possibility that resistance to gemcitabine might have been a consequence of an increased proportion of LRCC induced by gemcitabine treatment. It has been shown previously that cytotoxic chemotherapy, including gemcitabine in Panc1 cells, can induce stem cell fractions, as measured by side population fraction or by cell surface marker CD133 positive cell populations in pancreas cancer [32, 33]. Cy5^**+**^ labelled cells were expanded for a time period equal to six doubling times, and then treated with the GI50 dose of gemcitabine for two doubling times prior to sorting for LRCC and NLRCC. In the case of MiaPaCa2 and Panc-1, the average LRCC percentages after gemcitabine exposure was no different than without gemcitabine exposure (2.0% vs. 3.7%, *p* = 0.0746) (Fig. 3a).

In order to test the possibility that LRCC are quiescent relative to NLRCC and consequent decreased number of cell divisions confers resistance to gemcitabine therapy, cell cycle analysis of Cy5^**+**^ labelled cells were performed using a propidium iodide (PI) method. FACS analysis of PI staining revealed no difference in S-phase between LRCC and NLRCC of the average of the three cell lines (27.45% vs. 27.05%, *p* = 0.9247, Fig. 3b). Finally, to evaluate if gemcitabine resulted in slowed division of LRCC relative to NLRCC, cell cycle analysis was performed following gemcitabine exposure. Following exposure to GI50 gemcitabine concentrations there was also no difference in S-phase between LRCC and NLRCC (26.05% vs. 28.35%, *p* = 0.8006, Fig. 3c), nor was there any difference between the proportion of LRCC in S-phase which had been exposed to gemcitabine (26.05%) compared to the proportion of LRCC in S-phase (27.45%, *p* = 0.8893) of vehicle treated cells (Fig. 3d). These findings suggest mechanisms of resistance to gemcitabine are an indigenous feature of LRCC, not due to alterations in cell cycle progression or proliferation, and, at least initially, not associated with expansion of the LRCC fraction.

### Gene expression analysis

We next pursued possible intrinsic mechanisms of resistance inherent to the LRCC population by using a global comparative gene expression approach between the two cell populations. Unsupervised cluster analysis of gene expression levels of the LRCC, NLRCC and bulk cell fractions revealed that the LRCC and bulk fractions were more similar to each other than the NLRCC fraction for the cell lines Panc-1 and MiaPaCa2 on hierarchical clustering analysis visualized as dendrograms (data not shown). In each of the cell lines, the LRCC and NLRCC had the greatest distance between them by IPA dendrogram measurements considering both lengths of branches as well as the splits.

Probes representing genes up-regulated and down-regulated > 2.0-fold change in the LRCC fraction compared to the NLRCC fraction were identified. A Venn diagram analysis was constructed including all cell lines, which allowed for identification of the genes commonly up- or down-regulated common to all three lines. There were 383 probes up-regulated more than 2.0-fold change in the LRCC compared to the NLRCC and 432 probes down-regulated more than 2.0-fold change in the LRCC compared to the NLRCC.

**Fig. 4:**
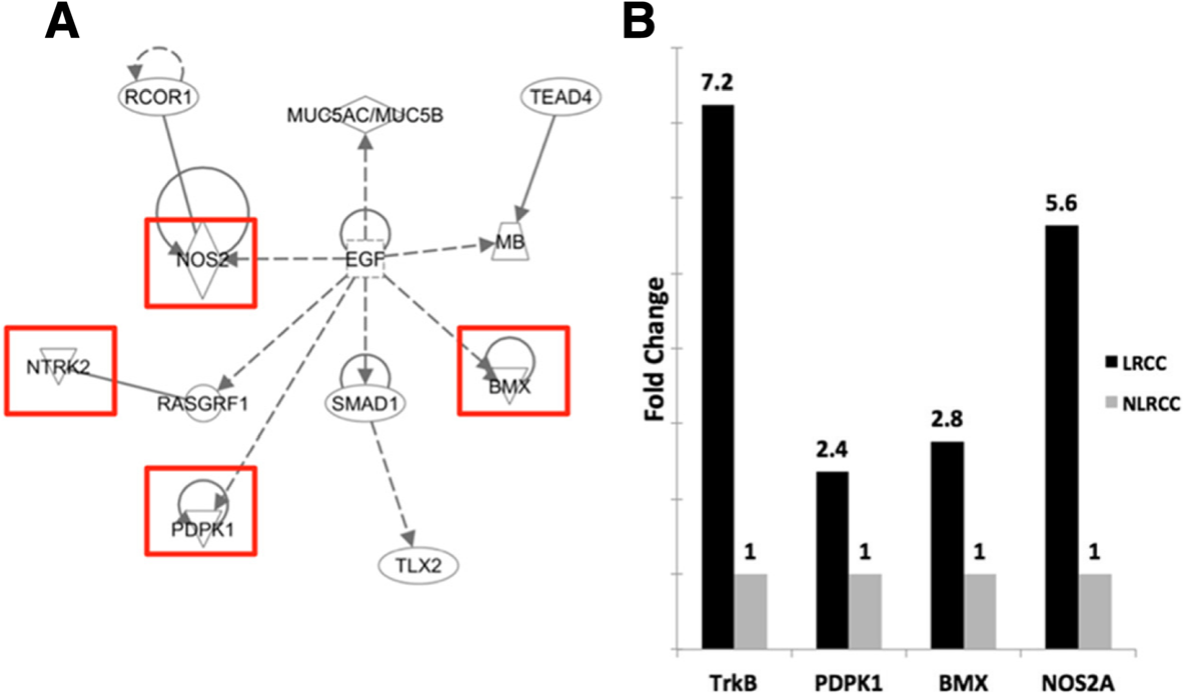
Differentially regulated genes in LRCCs with potential therapeutic value identified by **a**) Ingenuity Pathway Analysis (IPA). Only genes with a fold change > 2 and a FDR < 0.1 were considered, the highest scoring network is shown (genes selected for further analysis are highlighted). **b**) Validation of differential gene expression between LRCC and NLRCC cells identified on gene expression arrays by individual qRT-PCR

Pathway analysis of the up-regulated genes revealed enrichment of interactions around the EGF ligand mediated and related pathways as the top enriched network (Fig. 4a). Three kinases and a synthase were identified in this network—NOS2A (nitric oxide synthase 2; iNOS, Entrez ID 4843), NTRK2 (neurotrophic receptor tyrosine kinase 2; TrkB, Entrez ID 4915), PDPK1 (PDK1, Entrez ID 5170), and BMX (BMX non-receptor tyrosine kinase; ETK, Entrez ID 660). The average fold change of up-regulation in the LRCC compared to NLRCC across the three cell lines for each gene was 6.62, 2.74, 7.22, and 5.12 for NOS2A, NTRK2, PDPK1, and BMX, respectively). Validation of the microarray data using qRT-PCR confirmed expression fold change > 2.0 (LRCC vs. NLRCC) for each gene (NOS2A = 5.6, NTRK2 = 7.2, PDPK1 = 2.4, BMX = 2.8) initially observed on the microarray study (Fig. 4b).

### Response to gemcitabine following siRNA knockdown of target genes

**Fig. 5:**
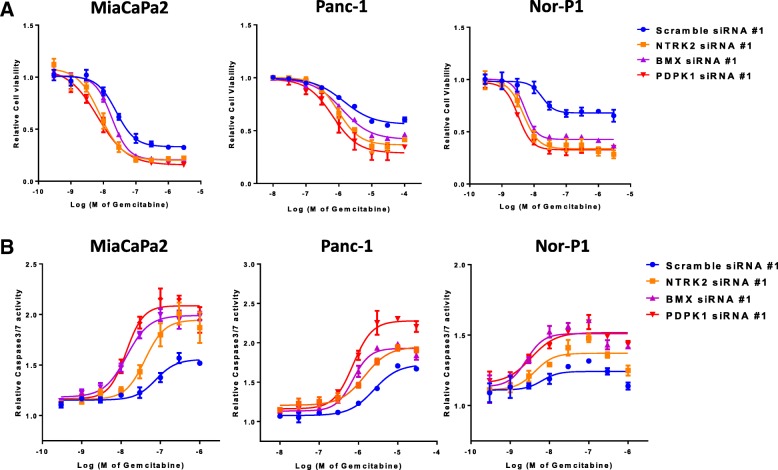
Silencing of BMX, NRTK2, and PDPK1 upregulated in LRCC increases response to gemcitabine in pancreas cancer cells. **a** anti-BMX, NRTK2 and PDPK1 siRNA leads to increased sensitivity to gemcitabine compared to cells with intact BMX, NRTK2 and PDPK1. Full drug response curves in MiaPaCa2, Panc-1, and Nor-P1 cells including cells transfected with scramble siRNA and indicated target siRNAs are shown. Gemcitabine concentration in logM on x-axis. **b** Induction of apoptosis in MiaPaCa2, Panc-1, and Nor-P1 cells treated with gemcitabine upon silencing of BMX, NRTK2, or PDPK1 (compared to scramble siRNA cells)

Next, to study whether these upregulated genes in the LRCC population are involved in mediation of response to gemcitabine, full drug-response curves of gemcitabine with and without silencing of the four tyrosine kinases overexpressed in the LRCC fraction were generated. First, four sequences for each target gene were tested for effective silencing of target genes confirmed by qRT-PCR in all cell lines and the two sequences inducing the most efficient loss of mRNA expression (≥88%) were selected for further studies (Fig. 5a, Additional file 1: Figure S1A and Additional file 1: Figure S1C). The reduction of knock-down of PDPK1 was also confirmed by Western Blot in all three cell lines (Additional file 1: Figure S1B). Drug response curves with gemcitabine in MiaPaCa2, Panc-1, and Nor-P1 cells (Fig. 5a and Additional file 1: Figure S1C) showed increased sensitivities to gemcitabine with IC50s greater than up to 10-fold lower compared to scrambled siRNA control upon silencing of BMX, NTRK2, and PDPK1. In line with reduced proliferation, silencing of the 3 genes in MiaPaCa2, Panc-1, and Nor-P1 cells significantly increased apoptosis upon treatment with gemcitabine (Fig. 5b**)**. In contrast, silencing of iNOS gene did not affect gemcitabine drug response.

**Fig. 6:**
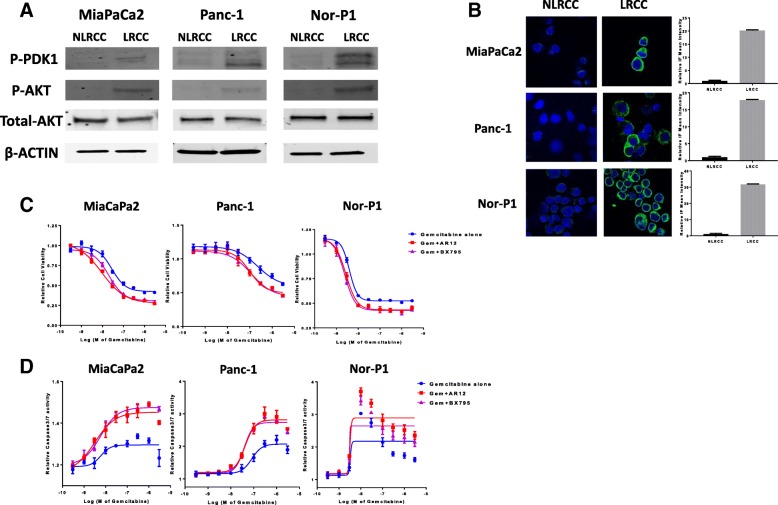
PDPK1 is activated in LRCC and silencing augments response to gemcitabine. **a** Immunoblots of LRCC and NLRCC fractions from MiaPaCa2, Panc-1, and Nor-P1 cells probed with anti-phospho PDPK1, anti-phospho AKT. Equal amounts of protein loaded, anti-AKT and β-Actin control on bottom. **b** Immunofluoresence of LRCC and NLRCC populations from MiaPaCa2, Panc-1, and Nor-P1 cells measuring anti-phosphatidylinositol 3,4,5-trisphosphate levels. Normalized mean of staining intensity for each population shown on the right. **c** Viability of pancreas cancer cells treated with gemcitabine alone (blue) or in combination with the PDPK1 inhibitor BX795 (purple) or AR-12 (red). **d** Induction of apoptosis in pancreas cancer cells treated with gemcitabine alone or in combination with the PDPK1 inhibitors BX795 and AR-12

Next, we investigated whether pharmacological inhibition could phenocopy above sensitization findings induced by siRNA silencing and selected PDPK1 as a major regulator for the activation of AGC kinases (serine/threonine kinases of the protein kinase A, G, and C family) and canonical AKT signaling in cancer cells. To show that LRCC also harbor increased PDPK1 activity, we first compared phospho-PDPK1 in the LRCC vs NLRCC fractions of the three cell lines next (Fig. 6a). In line with increased phospho-levels of PDPK1, levels of the upstream activator of PDPK1, phosphatidylinositol 3,4,5-trisphosphate (PIP3), required for membrane lipid binding and activation of PDPK1 for canonical AKT (AKT Serine/Threonine Kinase 1) activation was also significantly increased in LRCC vs NLRCC in all three cell lines (Fig. 6b). Next, we determined drug response profiles to the PDPK1 small molecule inhibitors BX795 and AR-12 in the three cell lines and selected concentrations of BX795 and AR-12 for gemcitabine combination studies which did not affect proliferation (Additional file 2: Figure S2). The addition of PDPK1 blockade increased the growth inhibitory effect of gemcitabine (Fig. 6c). In addition, the addition of the PDPK1 inhibitors to gemcitabine significantly increased the induction of apoptosis (Fig. 6d).

### PDPK1/PDK1 knockdown abrogates resistance to apoptosis in LRCC

**Fig. 7:**
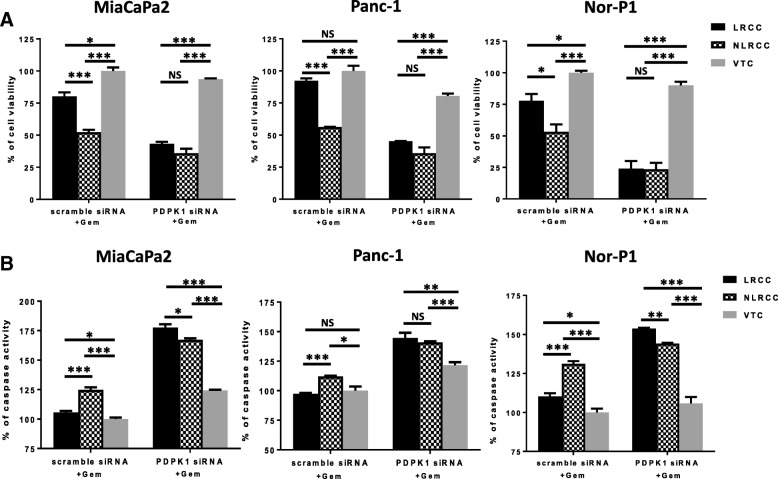
PDPK1 knockdown decreases resistance to gemcitabine in the LRCC population. **a** Proliferation of MiaPaCa2, Panc-1, and Nor-P1 cells exposed to gemcitabine at GI50 concentration for two doubling times and normalized to vehicle-treated when treated with scramble siRNA (left) and PDPK1 siRNA (right) (*p < 0.05, **p < 0.01, ***p < 0.001; paired *t*-test), and **b**) induced apoptosis measured by caspase3/7 levels

To provide support that knockdown of kinases with elevated expression levels in the slowly cycling LRCC fraction was indeed involved in the altered gemcitabine drug phenotype, we studied the impact of PDPK1 silencing in the individual LRCC and NLRCC cell subpopulation on cell growth and induction of apoptosis upon gemcitabine exposure in the three cell lines next. Following FACS of Cy5^**+**^ cells to separate LRCC and NLRCC, both cell populations were individually transfected with anti-PDPK1 siRNA. Equal knockdown in both cell populations was confirmed by qRT-PCR and viability and caspase levels of untreated cells transfected with scramble siRNA was set to 100%. In the absence of gemcitabine treatment there was minimal impact of loss of PDKP1 on cell viability or apoptosis levels in untreated cells when compared to cells transfected with scramble siRNA (Fig. 7a and b). In cells treated with gemcitabine, loss of PDPK1 affected growth of LRCC proportionally significantly more than the NLRCC fraction (% change compared to vehicle control cells of LRCC transfected with scramble siRNA vs PDPK1 siRNA: 80.23% vs 43.32% in MiaPaCa2, 92.28% vs 45.17% in Panc-1, and 77.88% vs 26.57% in Nor-P1) (Fig. 7a). Similarly, upon loss of PDPK1 gemcitabine, apoptosis levels more in LRCC compared to NLRCC (% change compared to vehicle control transfected with scramble siRNA vs PDPK1 siRNA: 105.64% vs 177.69% in MiaPaCa2, 97.32% vs 144.63% in Panc-1, and 110.26% vs 145.36% in Nor-P1) (Fig. 7b). In summary, these findings suggest PDPK1 signaling to be essential in LRCC for the mediation of gemcitabine resistance.

### PDPK1 is dysregulated in pancreatic cancer

**Fig. 8:**
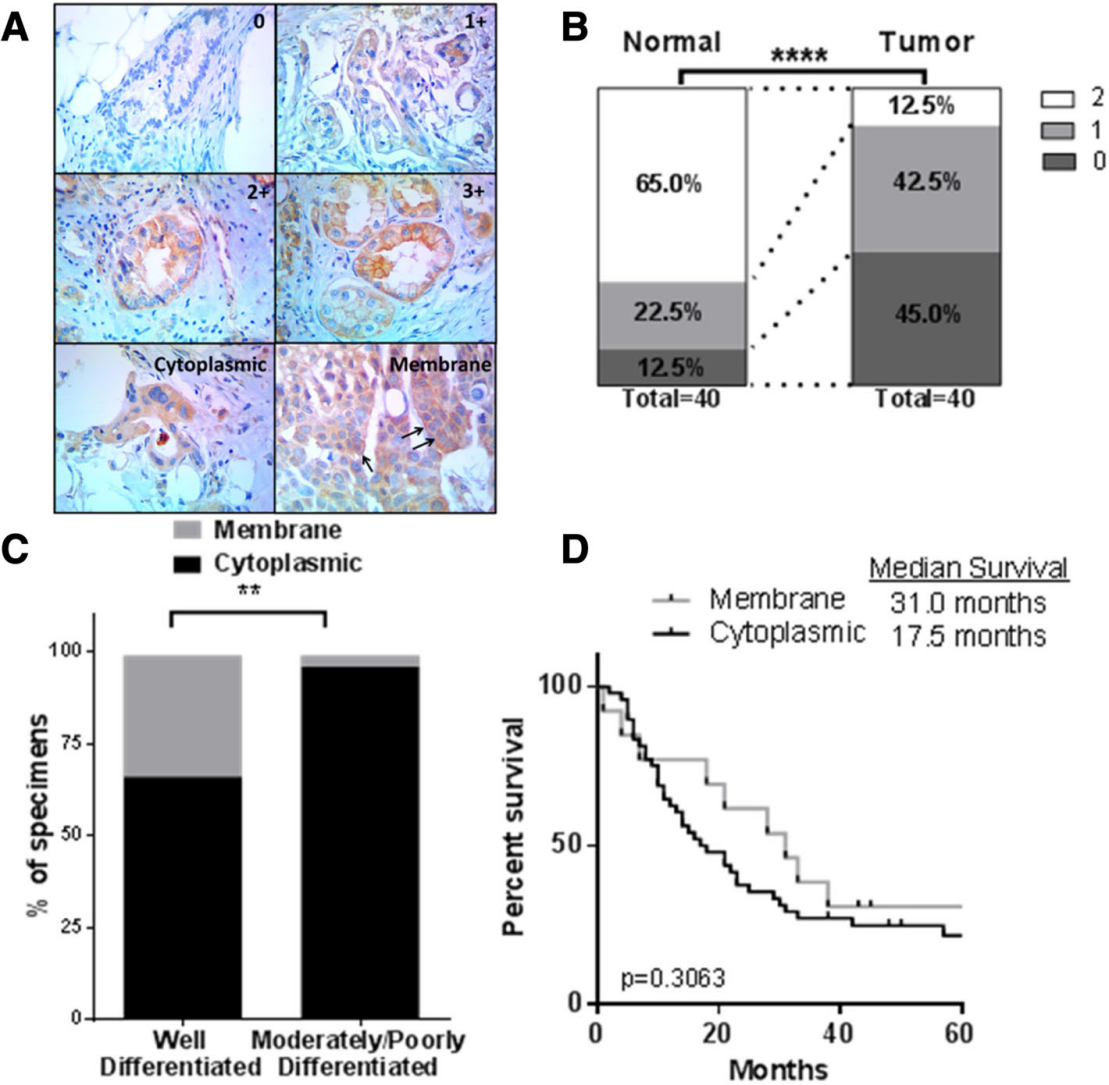
Comparison of PDPK1 expression in pancreatic tissue microarray. **a** Representative photomicrographs of pancreatic tissue microarray (TMA) cores illustrating expression levels of PDPK1 immunohistochemical staining (0,1+,2+,3+) and examples of cytoplasmic and membranous PDPK1 staining (arrows). **b** Reduced PDPK1 expression levels in tumor specimens compared to matched normal (uninvolved) pancreatic tissue (Wilcoxon matched-pairs signed rank test; *p* < 0.0001). **c** Membranous staining is more frequent in well-differentiated tumors (*p* = 0.0029, Fisher’s exact test). **d** Trend (log-rank test; *p* = 0.3063) towards improved survival in patients who showed membranous PDPK1 staining

To examine if correlative tissue studies on PDPK1 expression in clinical specimens support a role of this enzyme in pancreas cancer biology, we first examined levels of total PDPK1 expression in tumor versus matched normal, uninvolved pancreas tissue via IHC on a total of 40 pancreas cancer specimens. Significantly reduced PDPK1 levels were seen in the tumor specimens with a ≥ 5-fold reduction of the number of cases staining 2+ and a concomitant increase in the number of cases who lost PDPK1 or stained weakly positive in the tumors (Wilcoxon matched-pairs signed rank test; *p* < 0.0001) (Fig. 8a and b). We then examined association between PDPK1 expression levels and pathological variables and clinical outcome including overall survival. There was no association of overall expression with survival or correlation with one of the pathological variables nuclear grade, differentiation, or involvement of locoregional lymph nodes. Since a majority of studies on the potential role of PI3K-PDPK1-AKT signal transduction in a number of malignancies, including pancreas cancer, found that activation state measured as phosphorylation rather than amplification or overall expression levels to be clinically significant, we re-examined staining pattern of PDPK1 for membranous vs cytoplasmic staining. For PDPK1 to be activated by phosphatidylinositol (3,4,5)-trisphosphate (PIP3) generated by PI3K, its N-terminal pleckstrin homology domain provides a lipid-anchoring part to direct PDPK1 to PI3K-generated PIP3, recruiting the enzyme to the plasma membrane. Using membrane recruitment as a measure of PDPK1 activation, we compared membranous versus cytoplasmic staining patterns with nuclear grade distribution, lymph node involvement, and survival. Only ~ 15% of cases showed membranous staining. Membranous staining was negatively correlated with high nuclear grade (*p* = 0.0029, Fig. 8c) and there was a trend towards improved survival in patients who showed membranous PDPK1 staining in their surgical or biopsy specimens (Fig. 8d). Membranous staining was not associated with overall expression levels. These observed associations of perturbations of PDPK1 expression patterns in patients’ specimens suggest that PDPK1 regulation might be involved in pancreatic carcinogenesis and pancreas cancer biology.

## Discussion

Conventional treatment with systemic cytotoxic chemotherapeutics, which non-specifically targets rapidly dividing cells, is ideally replaced, or supplemented, by therapy that targets the cells driving recurrence and metastasis—the primary causes of most cancer-related death for patients afflicted by pancreas cancer [3]. Here we demonstrate that by inhibiting genes overexpressed in label-retaining cancer cells, an in vitro model of slowly cycling cells and subpopulation of CSC, can improve response to the standard cytotoxic chemotherapy with gemcitabine. The rationale of manipulating sub-populations of malignant cells within a tumor, in particular sub-populations involved in chemoresistance and metastasis, has recently been shown in elegant murine models of breast and pancreas cancer, and could have important implications in the treatment of patients with cancer [34–36].

While the identification of cancer stem cells in the literature is largely based on cell surface phenotype [12–14, 37–39], previous work from our group and others has shown that identification of a population of cells based on proposed function and hierarchy within a tumor is possible [19, 40]. Our group has previously shown that LRCC undergo asymmetric cell division with non-random chromosomal cosegregation (ACD-NRCC), which is consistent with the carcinogenesis (initiation and promotion) portion of the cancer stem cell theory [18, 20, 41]. Further, LRCC have been shown to be more tumorigenic, which is consistent with the tumor progression (tumor growth and metastasis) portion of the cancer stem cell theory [18, 25]. More recently, there have been more reports on LRCC mediating chemoresistance, early tumor recurrence, and metastasis [29, 42, 43]. However, there has been only one report on slowly cycling cells in pancreas cancer using the label Dil, and a surprising paucity on intracellular signaling features unique to LRCC governing chemoresistance or tumor initiation [44]. In this report we aimed to assess the possible clinical significance of the LRCC population in terms of response to gemcitabine, one of the standard therapies for patients with pancreas cancer. Deriving LRCC from different pancreatic cancer cell lines we first showed that LRCC are, in fact, resistant to this’therapeutic’ agent and that decreased rates of apoptosis upon gemcitabine treatment contributed to gemcitabine resistance phenotype of LRCC. One of the initially vexing findings was the decreased reduction of NLRCC growth after gemcitabine administration without a concomitant increase in the LRCC cells. We attributed this to the relative short treatment course of two doubling times possibly too short to detect differences considering the small number of LRCC but also possibly to a to-date incompletely understood interplay between LRCC and non-cancer stem cells along the concept of the ‘stem cell niche’. We speculate that LRCC might feed resistance signals to the bulk population but as viability and growth of NLRCC decreases as a consequence of the cytotoxic treatment ultimately also the pool of stem cells or LRCC is afflicted. Tumor resistance has then developed when stem cells recreate the niche, a biological function our presented in vitro LRCC model with limited gemcitabine exposure times was not able to measure. Such dynamic interplay between non-cancer stem cells and cancer stem cells has now been observed in a number of in vitro models and early paracrine signaling cues involved in this crosstalk involve HIFα (hypoxia inducible factors’ α subunits), sonic hedgehog, TGF**β**1 (transforming growth factor beta 1), and nodal/activin to name some described from pancreatic cancer stem cell niche models or, importantly, prostaglandin E2-induced repopulation of the tumor following chemotherapy treatment from the slowly cycling CSC as shown in patient-derived xenotransplanted bladder cancer [27, 45, 46].

In order to investigate differences between the two cell populations and possibly home in on mechanisms of action governing inherent chemoresistance features of the LRCC sub-population, we compared transcriptomic profiles of LRCC to the NLRCC population and identified a top-ranking network by IPA enriched for EGF ligand signaling. Of note, this network is different from the canonical erb receptor tyrosine kinase signal transduction cascades focusing on immediate EGFR (epidermal growth factor receptor) or HER2 (human epidermal growth factor receptor 2) downstream signaling studied in many cancers and more similar to finding from our prior work identifying EGF and MET-mediated signaling as top regulators of cell fate and lineage specific progenitor cell differentiation in the liver [41]. Studies by Takebe and Jeanes et al. in breast and other solid tumors on cell differentiation and stemness have also reported on an intimate role of a more extended, non-canonical erb signaling network and cancer stemness [47, 48]. We selected three kinases of the network for further loss-of-function studies and showed that NTRK2/TrkB, PDPK1/PDK1, and BMX/ETK were involved in regulation of gemcitabine sensitivity. Individual knockdown of PDPK1/PDK1 in both the LRCC and NLRCC subpopulations demonstrated that PDPK1/PDK1 inhibition lowered resistance to gemcitabine preferentially in the LRCC population and less in the NLRCC cells. Loss of PDPK1 had minimal impact on growth and apoptosis rates of untreated cells suggesting an essential role of this regulator in the LRCC population. Along these lines, recent work has PDPK1 also been implicated in the regulation of self-renewal, cellular transformation, and stemness in several diseases including cancer. Of importance, this work identified downstream signaling cascades regulated by PDPK1 outside the canonical receptor tyrosine kinase, PI3K and AKT-cascade PDKP1 was first described. These include phospholipase C, protein kinase C, or Hippo signaling governing stemness features through crosstalk with WNT [wingless-type MMTV (mouse mammary tumor virus) integration site] or β-catenin signaling [49–52].

In pancreas cancer, Eser et al. have shown that PDK1 is an essential effector of KRAS, and that an intact PDK1/PI3K axis is an essential tumor initiating event in cooperation with KRAS for increased cell plasticity, acinar-to-ductal metaplasia (ADM), and pancreatic ductal adenocarcinoma (PDAC) formation [53]. The pro-tumor function of an intact PI3K/PDPK1 axis reported by Eser and colleagues appears to be at odds with our findings of decreased PDPK1 expression levels in tumor tissues, and the associations of membranous localization with well-differentiated tumors and a trend towards improved clinical outcome. PDPK1 signals in a PI3K-depedent manner activating AKT, S6K (ribosomal protein S6 kinase), and SGK (serum/glucocorticoid regulated kinase) but can also activate PKC and p90RSK independent of PI3K and PIP3-mediated activation. PKC (protein kinase C) and p90RSK (p90 ribosomal S6 kinase) were found activated by alternative RAS-mediated signaling pathways independent of PDPK1 in the study by Eser et al. suggesting a non-essential role of PDPK1 in these tumors [53]. Additionally, downstream PDPK1 canonical signaling measured by phospho-AKT and phospho-GSK3β (S9) levels was lower in the evolved pancreas cancers compared to pre-cursor ADM and PanIN lesions also raising the possibility of the more involved cancers having become independent of the PDPK1 signaling axis and more driven by signal transduction perturbations of additional pathways acquired during the later stages of pancreas cancer progression [53]. Such a hypothesis appears to be in line with the decreased PDPK1 expression levels in the tumor vs matched clinical specimens on tissue microarray staining. While correlative tissue studies with phospho-AKT measures have shown the more commonly found negative correlation between increased AKT activation and clinical outcome, it is possible that there is a subset of pancreas cancer, similar to studies in non-small cell lung cancer, where phospho-AKT levels as a measure of EGFR-PI3K-AKT signaling have been shown to be associated with improved outcome [54–57]. It is intriguing to speculate that the hypothesis of a greater dependency on PDPK1 signaling in the early tumor-initiating events of ADM and PanIN formation, as seen in the transgenic animal studies, versus a later loss of addiction to canonical PDPK1 signaling and overtake by PDPK1 independent oncogenic events is commensurate with the cancer stem cell hallmark of tumor initiation by this cell population [12, 53]. Irrespective of possible differences in PDPK1 function in early vs late or primary vs metastatic tumors as a possible explanation of the observed PDPK1 expression pattern on our cancer tissue microarrays, the association of the heterogeneous PDPK1 expression pattern with important clinicopathological outcomes appears to be in line with the intratumoral heterogeneity of this regulator found to be overexpressed and activated in the LRCC vs NLRCC subpopulations where it is essential for chemoresistance.

On a preclinical level, PDPK1 has shown to confer oncogenic signaling and CSC renewal. The other targets derived from the differential gene expression screen in LRCC vs NLRCC have also been previously linked to stemness. The Tec kinase BMX non-receptor tyrosine kinase (BMX) has been shown a tumor promoting role in gliolastoma multiforme through mediation of self-renewal and growth, and the neurotrophic tyrosine receptor kinase 2 (NRTK2) in precursor growth and differentiation [14, 58, 59]. On the other hand, the gene expression differences identified in our in vitro model of cancer stemness, LRCC vs NRLCC, showed limited overlap with other in vitro models comparing gene expression and pathway alterations between stem cell and non-cancer stem cell fractions. For example, similar IPA network analysis comparing 3D spheroid Panc1 cells, originally described as the invasive Panc1 cell population, versus 2D monolayer cells resembling non-cancer stem cells identified as the top differentially regulated network genes involved in DNA damage repair [60]. When comparing side population versus non-side population bulk cells from patient-derived xenograft models an extensive network enriched with transcription factors of pluripotency, cell differentiation, and EMT (epithelial-mesenchymal transition) can be identified [61]. Whether these differences are due to the different platforms or due to the increasingly recognized heterogeneity of different CSC population is currently not known. The two currently available drug discovery studies performed in pancreas cancer stem cell in vitro models using either a reporter line for the 26S proteasome activity of pancreas cancer cells or a 3D pancreatic carcinoma spheroid model yielded drug activities in the 3D CSC model with inhibition of phosphoinositide 3-kinase signaling, however there was an underrepresentation of selective PDPK1 inhibitors [62, 63].

This study is not without its limitations. First, the isolation of LRCC requires the assumption that all cells within the Cy5^+^ labeled population are expanding at the same rate. Since long term cultured cell lines were used, this assumption is based on the clonality and homogeneity of these lines. While cells were synchronized via transfer to serum-free media 24 h prior to labeling we cannot rule out that present clonal subpopulation with different growth rates in the used pancreas cancer cell lines increased the NLRCC subpopulation. However, we believe this fraction to be small. Second, the apoptosis experiments upon administration of gemcitabine were based on the concentration of the drug for 50% of maximal inhibition of cell proliferation, GI50, after two doubling times (determined individually for each cell line). This includes experiments on induction of apoptosis upon silencing the genes found to be overexpressed in the LRCC population. We cannot rule out that the impact on induction of apoptosis, or silencing of PDPK1 on gemcitabine drug response, might have been higher or lower either in the LRCC or NLRCC populations when other concentrations were used. For the presented in vitro comparisons, it was necessary to establish a consistent cutoff point that allowed for comparisons including downstream evaluation (i.e. qRT-PCR, cell cycle analysis, FACS) without complete mortality of the cells after gemcitabine exposure.

## Conclusion

In summary, we present in LRCC, an in vitro model of slowly cycling pancreatic cancer stem cells implicated in chemoresistance and tumor recurrence, the discovery of novel targets involved in response to gemcitabine treatment, one of the standard chemotherapy approaches used for pancreas cancer in the clinic. For decades, chemotherapy regimens have been based on the cell cycle differential that exists between normal and malignant tissue. Given the continued and unchanged dismal prognosis for patients with pancreas cancer this rationale has proven not specific enough and not sufficiently efficacious. Here, we have shown that LRCC harbor distinct transcriptomic profiles involved in mediation of chemoresistance and that targeting essential regulators of this program in LRCC can sensitize pancreas cancer cells. These findings offer novel hypotheses for the derivation of effective combination therapy approaches in a disease void of impactful interventions.

## Additional files

Additional file 1: **Figure S1.** Confirmation of silencing of BMX, NRTK2, and PDPK1 mediated by siRNA (set 1- #1) knockdown. A) Expression level of BMX, NRTK2 and PDPK1 in in MiaPaCa2, Panc-1, and Nor-P1 cells when scramble siRNA or anti- BMX, NRTK2 and PDPK1 siRNA (set 1- #1) are present. B) Protein level of BMX, NRTK2 and PDPK1 of the three cell lines in immunoblotting. C) anti-BMX, NRTK2 and PDPK1 siRNA (set 2- #2) leads to increased sensitivity to gemcitabine compared to cells with intact BMX, NRTK2 and PDPK1. Full drug response curves in MiaPaCa2, Panc-1, and Nor-P1 cells including cells transfected with scramble siRNA and indicated target siRNAs (set 2- #2) are shown. (PPTX 652 kb)

Additional file 2: **Figure S2.** Full drug response curves of the PDPK1 inhibitor BX795 (purple, A) or AR-12 (red, B) in MiaPaCa2, Panc-1, and Nor-P1 cells. (PPTX 510 kb)

## Abbreviations

ACD-NRCC: Asymmetric cell division with non-random chromosomal cosegregation
ADM: Acinar-to-ductal metaplasia
AGC Kinases: serine/threonine kinases of the protein kinase A, G, and C family
AKT: AKT Serine/Threonine Kinase 1
BMX: BMX non-receptor tyrosine kinase
CSC: Cycling cancer stem cells
EGF: Epidermal growth factor
EGFR: Epidermal growth factor receptor
EMT: Epithelial-mesenchymal transition
HER2: Human epidermal growth factor receptor 2 (receptor tyrosine-protein kinase erbB-2)
HIFα: Hypoxia inducible factors’ α subunits
LRC: Label retaining cells
LRCC: Label-retaining cancer cells
NOS2A: NOS2 nitric oxide synthase 2
NTRK2: Neurotrophic receptor tyrosine kinase 2
P90RSK: p90 ribosomal S6 kinase
PDAC: Pancreatic ductal adenocarcinoma
PDPK1 (PDK1): 3-phosphoinositide dependent protein kinase-1
PI: Propidium iodide
PIP3: Phosphatidylinositol 3,4,5-trisphosphate
PKC: Protein kinase C
S6K: Ribosomal Protein S6 Kinase
SGK: Serum/glucocorticoid regulated kinase
TGFβ: Transforming growth factor beta
TMA: Tissue microarray
WNT: Wingless-type MMTV (mouse mammary tumor virus) integration site
