# Supplementary material for: Loss of PDPK1 abrogates resistance to gemcitabine in label-retaining pancreatic cancer cells
Source: BMC Cancer. 2018 Jul 31;18:772. doi: 10.1186/s12885-018-4690-1 (PMC6069886; doi:10.1186/s12885-018-4690-1)
Supplement: Supplementary file 1 — Figure S1. Confirmation of silencing of BMX, NRTK2, and PDPK1 mediated by siRNA (set 1- #1) knockdown. A) Expression level of BMX, NRTK2 and PDPK1 in in MiaPaCa2, Panc-1, and Nor-P1 cells when scramble siRNA or anti- BMX, NRTK2 and PDPK1 siRNA (set 1- #1) are present. B) Protein level of BMX, NRTK2 and PDPK1 of the three cell lines in immunoblotting. C) anti-BMX, NRTK2 and PDPK1 siRNA (set 2- #2) leads to increased sensitivity to gemcitabine compared to cells with intact BMX, NRTK2 and PDPK1. Full drug response curves in MiaPaCa2, Panc-1, and Nor-P1 cells including cells transfected with scramble siRNA and indicated target siRNAs (set 2- #2) are shown. (PPTX 652 kb) [file 12885_2018_4690_MOESM1_ESM.pptx]

## Slide 1
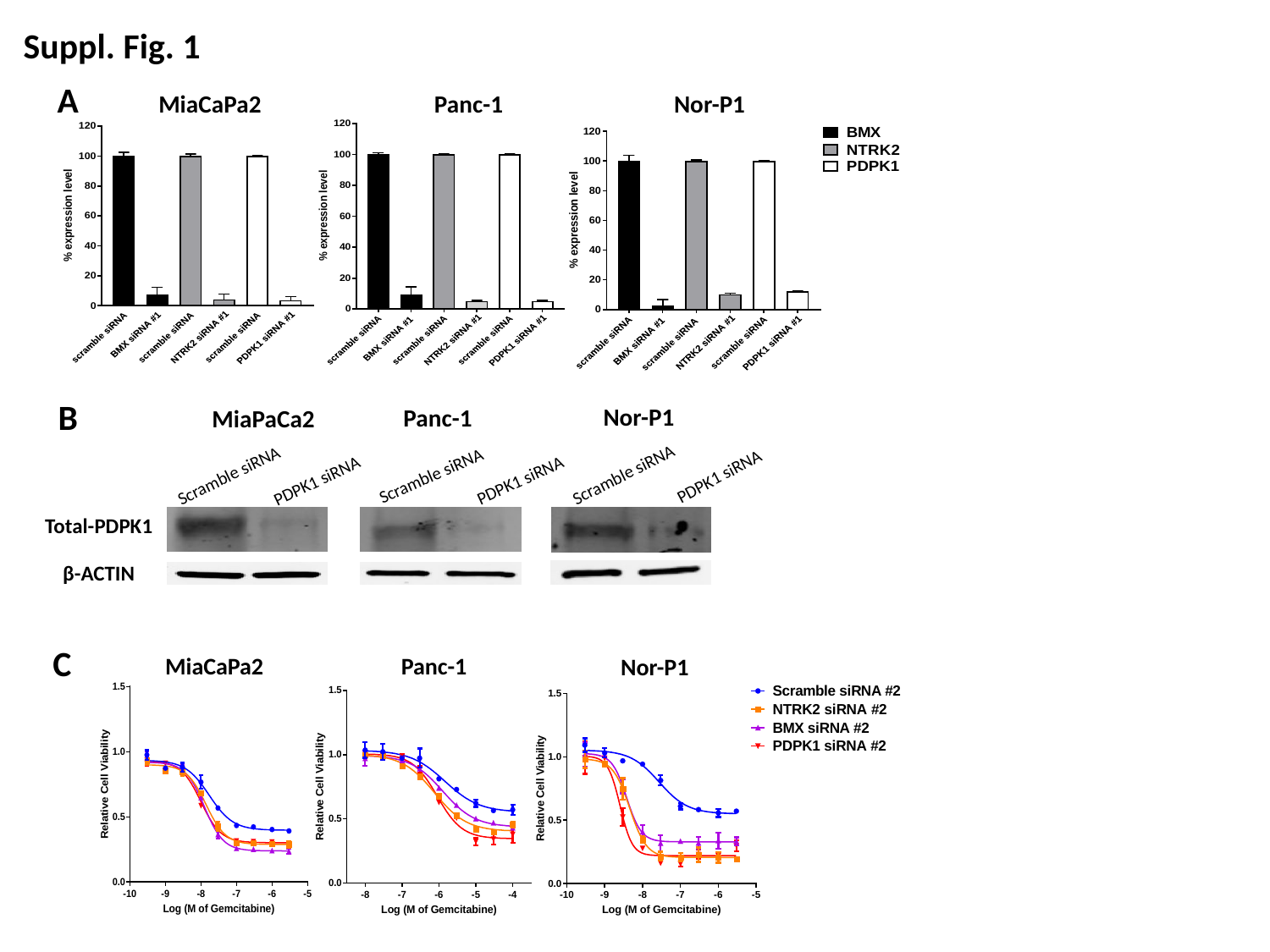

# Suppl. Fig. 1
A
MiaCaPa2
Panc-1
Nor-P1
B
Nor-P1
Panc-1
MiaPaCa2
Scramble siRNA
Scramble siRNA
Scramble siRNA
PDPK1 siRNA
PDPK1 siRNA
PDPK1 siRNA
Total-PDPK1
β-ACTIN
C
MiaCaPa2
Panc-1
Nor-P1
