# Supplementary figures and images for: Loss of PDPK1 abrogates resistance to gemcitabine in label-retaining pancreatic cancer cells
Source: BMC Cancer. 2018 Jul 31;18:772. doi: 10.1186/s12885-018-4690-1 (PMC6069886; doi:10.1186/s12885-018-4690-1)

## Slide 1
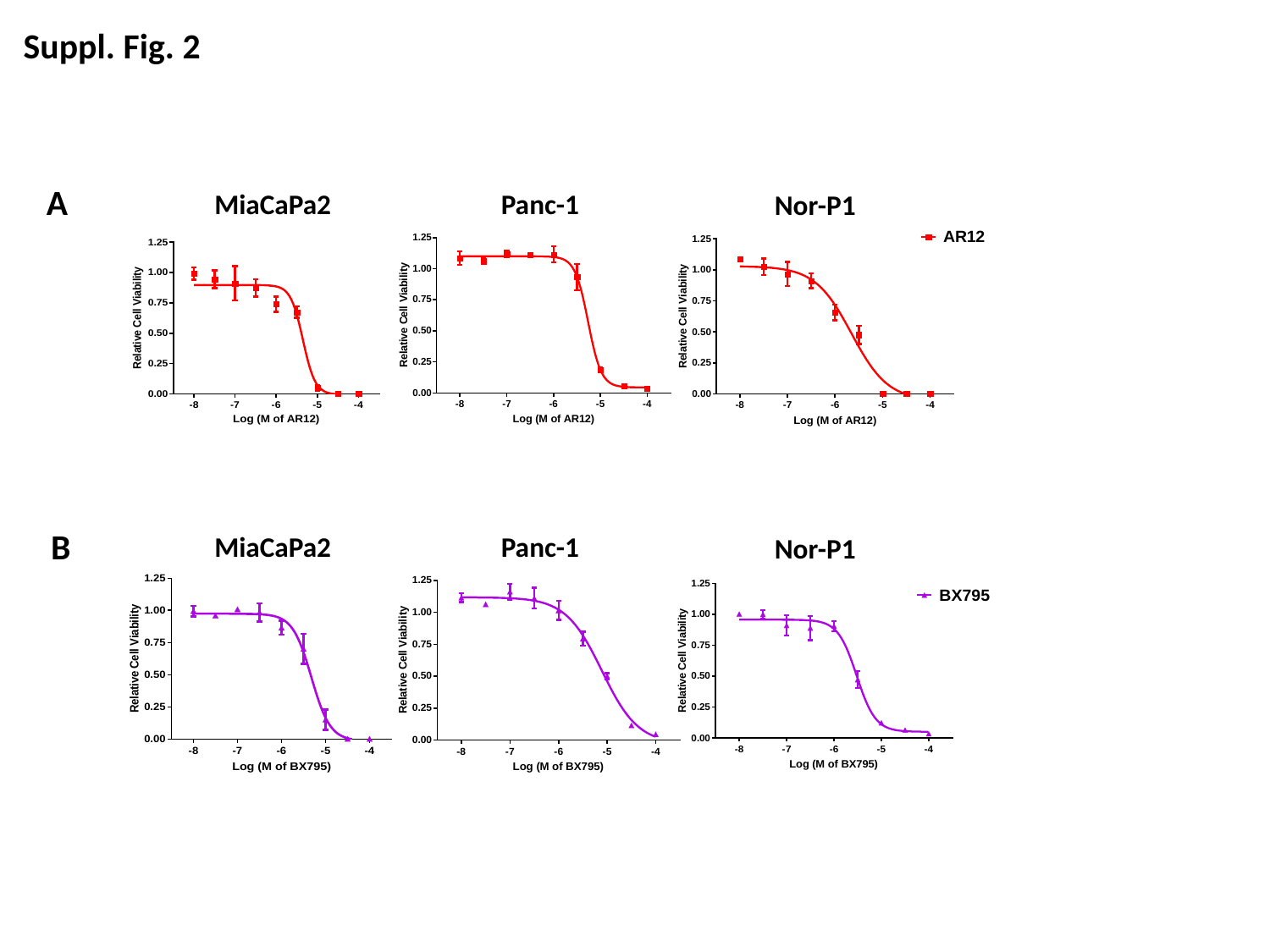

# Suppl. Fig. 2
A
MiaCaPa2
Panc-1
Nor-P1
B
MiaCaPa2
Panc-1
Nor-P1

Supplement: Supplementary file 2 — Figure S2. Full drug response curves of the PDPK1 inhibitor BX795 (purple, A) or AR-12 (red, B) in MiaPaCa2, Panc-1, and Nor-P1 cells. (PPTX 510 kb) [file 12885_2018_4690_MOESM2_ESM.pptx]
